# Large language models as decision aids in neuro-oncology: a review of shared decision-making applications

**DOI:** 10.1007/s00432-024-05673-x

**Published:** 2024-03-19

**Authors:** Aaron Lawson McLean, Yonghui Wu, Anna C. Lawson McLean, Vagelis Hristidis

**Affiliations:** 1 Department of Neurosurgery, Jena University Hospital–Friedrich Schiller University Jena, Am Klinikum 1, 07747 Jena, Germany; 2 Comprehensive Cancer Center Central Germany, Jena, Germany; 3 Department of Health Outcomes and Biomedical Informatics, College of Medicine, University of Florida, Gainesville, FL USA; 4 Computer Science and Engineering, University of California, Riverside, Riverside, CA USA

**Keywords:** Shared decision making, Large language models, Neuro-oncology care, Patient engagement, Ethical considerations, Healthcare integration

## Abstract

Shared decision-making (SDM) is crucial in neuro-oncology, fostering collaborations between patients and healthcare professionals to navigate treatment options. However, the complexity of neuro-oncological conditions and the cognitive and emotional burdens on patients present significant barriers to achieving effective SDM. This discussion explores the potential of large language models (LLMs) such as OpenAI's ChatGPT and Google's Bard to overcome these barriers, offering a means to enhance patient understanding and engagement in their care. LLMs, by providing accessible, personalized information, could support but not supplant the critical insights of healthcare professionals. The hypothesis suggests that patients, better informed through LLMs, may participate more actively in their treatment choices. Integrating LLMs into neuro-oncology requires navigating ethical considerations, including safeguarding patient data and ensuring informed consent, alongside the judicious use of AI technologies. Future efforts should focus on establishing ethical guidelines, adapting healthcare workflows, promoting patient-oriented research, and developing training programs for clinicians on the use of LLMs. Continuous evaluation of LLM applications will be vital to maintain their effectiveness and alignment with patient needs. Ultimately, this exploration contends that the thoughtful integration of LLMs into SDM processes could significantly enhance patient involvement and strengthen the patient-physician relationship in neuro-oncology care.

## Introduction

Recent years have witnessed a significant shift in the approach to surgical consent and treatment planning, moving away from traditional medical paternalism towards shared decision-making (SDM) (Edwards et al. 2023). SDM is an ethical and professional approach that fosters a collaborative patient-physician relationship, emphasizing the importance of informed consent based on mutual understanding and consideration of the best available evidence (Légaré et al. 2018). This dynamic process of SDM aims to improve health outcomes and enhance patient satisfaction by involving patients in decision-making processes (Mik et al. 2018; Elwyn et al. 2016).

## Implementation of SDM in neuro-oncology

In neuro-oncology, SDM is recognized as a crucial component in treatment decisions, aiming to involve patients and physicians in collaborative discussions about treatment and goals of care (Corell et al. 2021; Musella et al. 2021). Unique to neuro-oncology, compared to other healthcare domains, are the specific challenges of SDM, such as the complex nature of the disease, the urgency of treatment decisions, cognitive challenges faced by patients, and the multifaceted treatment options available (Díaz et al. 2009; Shepherd et al. 2023). These distinct challenges, coupled with barriers on both the patient and physician sides, can hinder the effective implementation of SDM in neuro-oncology care (Leu et al. 2023). Addressing these barriers is crucial to ensure that SDM interventions truly achieve their intended goals of equitable education and emotional support for all stakeholders involved (Waddell et al. 2021).

In this article, we examine challenges impeding the implementation of SDM in neuro-oncology and explore the potential of conversational artificial intelligence (AI), exemplified by large language models (LLMs) such as OpenAI’s ChatGPT and Google's Bard, to overcome these challenges. We argue that LLMs, sophisticated computational tools that generate coherent and contextually appropriate text responses from extensive datasets, may hold distinctive benefits in addressing these challenges. To examine this perspective, we undertook a thematic analysis, drawing insights from a diverse range of literature spanning academic databases, tech-centric repositories such as arXiv and IEEE Xplore, preprint platforms such as bioRxiv and medRxiv, and other research aggregators (Roberts et al. 2019). Our multifaceted approach sought to provide a comprehensive and nuanced view of SDM's intricacies in the neuro-oncology landscape and the potential role of LLMs in overcoming these hurdles.

## Barriers on the patient side

Patients diagnosed with neuro-oncological conditions often face numerous challenges in understanding and decision-making. Foremost among these is limited health literacy (Chieffo et al. 2023; Porter et al. 2021; Reinert et al. 2018). The medical world, laden with its complex terminology and detailed information, can be intimidating (Koch-Weser et al. 2009). Many neuro-oncology patients, despite earnest efforts, find themselves overwhelmed and unable to fully understand their treatment options, hindering their capacity to make informed decisions (Sorensen Von Essen et al. 2022b). There is an urgent need for new communications strategies that effectively bridge this gap in health literacy and patient self-efficacy.

In addition, the profound emotional impact of a neuro-oncological diagnosis can overshadow a patient's ability to communicate and make clear informed decisions. Emotions such as fear, anxiety, and overwhelming uncertainty can prevent them from actively participating in SDM discussions (Hermann et al. 2016). Recent studies by Leu et al. 2023; Sorensen Von Essen et al. 2022 underscore the importance of not only medical guidance but also emotional support in the neuro-oncology context (Leu et al. 2023; Sorensen Von Essen et al. 2022a). Creating a safe environment for patients to voice their fears and concerns is vital in alleviating the burden of these emotional obstacles (Heyhoe et al. 2015).

Furthermore, conditions such as brain tumors often result in cognitive impairments. These mental alterations hinder the patient's capacity to process complex medical information and engage actively in SDM (Coomans et al. 2019; Gerstenecker et al. 2014; Gosselt et al. 2021). Evaluating cognitive functions in such a situation becomes essential. Involving caregivers or relatives in the decision-making process is also crucial (Pace et al. 2020). It is worth highlighting that the caregivers or close family members must also navigate a turbulent emotional landscape, often mirroring the confusion, fear, and lack of understanding experienced by the patients themselves (Hewins et al. 2019; CaC and Broekman 2022).

In today's information-rich age, there is another prevalent challenge: information overload. Patients and caregivers are often swamped by the sheer volume of information available, especially on the internet (Sorensen Von Essen et al. 2022b). This information overload can lead to decision paralysis, making it hard for them to discern and understand treatment options and potential outcomes. It is crucial for healthcare providers and patients to be aware of these barriers and identify strategies to address them together.

## Barriers on the physician side

Physicians, in their commitment to deliver the best neuro-oncology care, face a set of distinct challenges, which may affect the implementation of SDM.

One of the most pervasive challenges is time constraints. With busy schedules and ever-increasing patient loads, physicians often find themselves pressed for time and even burned out (Downing et al. 2018; Kroth et al. 2019). Unfortunately, these limited windows for consultation can hinder the depth and quality of discussions and SDM. Elwyn et al. stress the implications of this limited engagement, highlighting the need for allocating adequate time to ensure enriching patient-physician interactions, fundamental for a holistic SDM approach in neuro-oncology care (Elwyn et al. 2016).

Within the challenges of SDM in neuro-oncology, one cannot overlook the profound emotional toll and associated burnout that physicians often grapple with. In a survey of European and North American neuro-oncology providers conducted in 2016–2017, it was observed that professional burnout is a significant issue among those dedicated to the care of patients with brain tumors (Yust-Katz and O’brien B et al. 2020). This syndrome, characterized by emotional exhaustion, depersonalization, and a sense of diminished personal achievement, underscores a silent crisis among healthcare providers in the realm of neuro-oncologist (Dunbar and Kumthekar 2020). The implications of such burnout cannot be understated, especially when deliberating on SDM's effectiveness in neuro-oncology. An emotionally drained physician, grappling with burnout, might not optimally engage in shared decision-making, potentially sidelining the patient's perspective (West et al. 2018). Addressing physician burnout, therefore, becomes key not just for the well-being of healthcare providers but also for the overarching efficacy of the SDM paradigm in neuro-oncology care.

Next comes the challenge of staying updated in a rapidly evolving field. The swift pace of advancements in neuro-oncological research and treatment modalities can make it difficult for physicians to keep up to date (Lukas et al. 2018). Any knowledge gaps, however, slight, could compromise their ability to provide the most recent and comprehensive information to patients, thereby affecting the SDM process. This demonstrates the importance of continuous medical education and easy access to trustworthy resources, which can help physicians provide a consistently high standard of care.

Historically, the physician–patient relationship has often been asymmetrical, with the former assuming a more dominant role (Kaba and Sooriakumaran 2007). This imbalance, coupled with ingrained biases, can sometimes tilt the scales away from effective SDM (Makoul and Clayman 2006). A tendency towards paternalistic decision-making can lead to decisions being made without fully integrating the patient's perspective. Edwards et al. 2023 identified the significance of recognizing and addressing these biases in the context of SDM involving patients with advanced cancer deciding on palliative treatments and care (Edwards et al. 2023). A transition towards truly patient-centered care, where the patient's voice is equally resonant, becomes crucial for the effective implementation of SDM.

Finally, even with the best intentions, effective communication remains an elusive skill for some. While medical knowledge is vast, conveying it without resorting to jargon or overly complex language is vital. Any misunderstanding in communication can create barriers to patients’ understanding, depriving them of a meaningful role in the decision-making process (Vermeir et al. 2015). Therefore, mastering clear and accessible communication techniques is essential. By ensuring that complex information is relayed in an understandable manner, physicians empower patients to become active participants in their own care journey. In sum, while the challenges faced by physicians in the realm of SDM are multifaceted, acknowledging and proactively addressing them can pave the way for more collaborative and informed neuro-oncology care.

## Bridging the gap with LLMs

LLMs might offer some assistance in enhancing patient education, counseling, and SDM within the complex field of neuro-oncology care. It is necessary to approach their potential with measured optimism, recognizing their limitations while considering their potential advantages for disseminating information and bolstering patient engagement (Ray 2023).

LLMs have the capability to provide informative responses tailored to one-off or conversational interactions with users, based on the knowledge that the LLMs have acquired through analyzing millions of documents, a process known as “training”. LLMs can potentially aid in clarifying medical notions, laying out and explaining treatment options, and conversing about possible pros and cons in a comprehensible way (Singhal et al. 2023). With a potential basis in clinical evidence, LLMs might offer patients a clearer lens through which to view their care choices, and how these align with their personal objectives (Jin et al. 2021). Although they generally do not have access real-time patient specifics, the immense computational power of LLMs can still be looked at as a resource for broad knowledge sharing, possibly making medical concepts more understandable for patients (Harrer 2023).

In relation to SDM, LLMs could potentially be utilized as decision-support tools, presenting patients with overarching views of treatment alternatives (Yang et al. 2023). By tapping into an expansive knowledge sourced from neuro-oncology cases, clinical standards, scientific findings, and clinical trial data, LLMs might address patient queries, mitigate apprehensions, and generally improve understanding. LLMs might also help patients prepare for their next physician visit, by jointly creating a small set of questions to ask their healthcare provider. With the right kind of information, patients might find themselves in a better position to partake in decision-making processes.

One significant advantage of LLMs over conventional web search engines, such as Google, is their capacity to alleviate the issue of information overload. Rather than presenting users with an overwhelming array of web pages, LLMs synthesize information into concise, coherent paragraphs that respond directly to the user's query, thereby facilitating patient education and setting the stage for more substantive discussions within the framework of SDM For LLMs to effectively contribute to SDM, it is imperative that the information they provide is accessible to patients, necessitating a literacy level commensurate with that of the intended audience. Recent analyses have indicated that outputs from models like ChatGPT often demand high literacy levels for comprehension, posing challenges in the context of patient education on complex medical topics (Dash et al. 2023; Haver et al. 2024; Onder et al. 2024; Temel et al. 2024). However, this limitation can be addressed through strategic prompting—the method by which a query is formulated by the user (Gao 2023). For instance, a user might specify, “Explain low-grade glioma as if to a 10th-grader,” thus tailoring the complexity of the LLM’s response to suit the patient's needs.

It is critical to emphasize that current LLMs do not possess the requisite maturity for direct healthcare applications, and thus, should not be considered replacements for healthcare professionals (Dinan et al. 2021). Rather, they function as supplementary informational aids that can enhance patient education and facilitate more comprehensive discussions. A principal limitation preventing LLMs from acting as substitutes for physicians is the lack of guaranteed scientific accuracy in their responses (Mittelstadt et al. 2023). This inaccuracy arises not only from potential misinformation present in the training datasets but also from the phenomenon known as “hallucination,” where an LLM generates content that is not verifiably sourced from its training data (Huang et al. 2023). Addressing this issue of hallucination represents a significant focus of ongoing research.

Furthermore, LLMs may not deliver the most current or advanced information due to delays in retraining cycles, which occur infrequently due to high computational costs (Ling et al. 2023). For instance, updates to models such as ChatGPT are not continuous, resulting in responses that may lack the latest scientific findings or treatment advancements. This temporal gap in knowledge underscores the models' limitations in providing real-time, state-of-the-art medical advice. Despite these constraints, the application of LLMs in patient education offers the potential to alleviate the burden of information overload on healthcare practitioners, thereby enabling them to dedicate more attention to patient-centered care, consider the clinical implications of medical information, and offer personalized advice. This strategic utilization of LLMs could play a crucial role in enhancing the quality of healthcare delivery by supporting the informational needs of patients and practitioners alike.

The integration of LLMs for SDM in neuro-oncology is detailed in the workflow show in Table 1, beginning with patient input and concluding with the process's evaluation and refinement. Illustrating this approach with a young patient diagnosed with a low-grade glioma highlights the capability of LLMs to enrich each stage of care. While this offers guide for incorporating LLMs into neuro-oncology SDM, practical implementations may vary to suit distinct healthcare environments and resource availability.

**Table 1:** Workflow for utilizing LLMs in shared decision-making for neuro-oncology care

Step	Description
Step 1: Patient input	Patient provides information, concerns, and preferences regarding their condition and treatment options Examples from the case of a young patient diagnosed with a low-grade glioma following a brain biopsy: What is a low-grade glioma? What are my treatment options for a low-grade glioma? What are the potential risks and benefits associated with each treatment option? How will treatment for a low-grade glioma affect my daily life and long-term outcomes?
Step 2: LLM interaction	LLM interacts with the patient, generating tailored responses to address patient queries and provide relevant information
Step 3: Information dissemination	LLM presents evidence-based information about treatment options, including risks, benefits, and potential outcomes
Step 4: Patient consideration	Patient reviews the information provided by the LLM, considers their preferences and priorities, and explores treatment possibilities
Step 5: Patient-physician discussion	Patient engages in a discussion with the physician, sharing insights gained from the LLM interaction and discussing treatment options in more detail
Step 6: Physician interpretation	Physician analyzes patient-specific data, interprets clinical implications, and provides personalized advice and recommendations
Step 7: Post-discussion LLM interactio	After the patient-physician discussion, the patient interacts again with the LLM to clarify any remaining questions or concerns regarding the physician's recommendations. This step aims to reinforce understanding and ensure the patient feels fully informed
Step 8: Collaborative decision-making	Patient and physician collaborate in making informed decisions, taking into account the patient's values, preferences, and clinical expertise
Step 9: Follow-up and evaluation	Treatment decisions are implemented, and the patient’s progress is monitored. The effectiveness of LLM-based SDM is evaluated and refined for future improvements

This table presents an advanced framework for incorporating LLMs into the treatment decision-making process for patients with low-grade gliomas. It delineates a sequential, nine-step pathway that begins with the patient's initial input about their condition and concludes with the follow-up and evaluation of the chosen treatment. The structure emphasizes the integration of AI tools in healthcare, aiming to enhance patient engagement, satisfaction, and outcomes by providing tailored, accessible information throughout the decision-making journey. The specific implementation may vary depending on the context and available resources

*AI* artificial intelligence, *LLM* large language model

### Ethical considerations

The burgeoning integration of LLMs in healthcare, particularly in the domain of neuro-oncology, brings with it a host of ethical challenges. As these AI-driven tools increasingly influence patient education, counseling, and SDM, we must carefully examine a series of ethical considerations to chart a responsible and effective future for this technology.

First, ensuring patient privacy and informed consent is paramount when integrating LLMs into healthcare. LLMs process individual patient data to generate personalized responses, necessitating rigorous data protection measures, anonymization techniques, and comprehensive informed consent frameworks to safeguard patient confidentiality while leveraging LLM capabilities (Meskó and Topol 2023). Recent incidents, such as ChatGPT’s inadvertent leakage of sensitive business information belonging to a private company, underscore the imperative for scrutinizing the security and potential risks associated with LLM use (Borger et al. 2023; Nasr et al. 2023).

The feasibility of patients using public LLM platforms to discuss health conditions without compromising privacy remains problematic. Even without disclosing identifiable information, research has demonstrated the potential for deducing user identities through aggregated web search queries (Hussien et al. 2013; Zhang et al. 2022). One proposed solution is the deployment of private, locally hosted LLMs, which, while addressing privacy concerns, introduces significant costs associated with maintaining the necessary computational infrastructure (Hong et al. 2023). Additionally, such private models may lag behind the continuous advancements achieved by publicly available LLM technologies, presenting a trade-off between privacy protection and access to cutting-edge capabilities.

The responsible use of these AI-driven tools stands as another cardinal consideration. As they become more entrenched in our clinical workflows, it's vital to anchor their deployment in established clinical guidelines, evidence-based approaches, and professional standards (Benjamens et al. 2020; Topol 2019). This necessitates that LLM-informed chatbots and decision aids are not just technologically sophisticated but are also grounded in rigorous scientific scrutiny (Li et al. 2024; Sallam 2023b). For professionals to have faith in the outputs of LLMs, there must be transparent documentation detailing the algorithms’ inner workings and the data on which they were trained (Liao and Wortman Vaughan 2023).

Moreover, the journey with LLMs doesn’t end at their integration; it demands continual evaluation and refinement (Mökander et al. 2023). Ensuring these tools serve the patient optimally requires vigilance against potential biases and a commitment to enhancing their efficacy. By regularly analyzing their outputs and absorbing user feedback, we can sharpen their utility (Lee et al. 2023). For example, the recently developed concept of retrieval-augmented generation (RAG) has emerged as an avenue to enhance the specificity and relevance of LLM responses. By embedding updated clinical data and trusted medical sources, the RAG approach promises to provide more tailored prompt responses and guidance (Zakka et al. 2024; Wang et al. 2023). The endeavor to refine LLMs in neuro-oncology care must be a collaborative one, pooling insights from clinicians, researchers, and, crucially, the patients themselves.

Finally, while LLMs stand as a testament to technological prowess, it's vital to delineate their role clearly. They are instruments of information and guidance, not replacements for the nuanced care of healthcare professionals (Baumgartner and Baumgartner 2023; Bommasani et al. 2023). Physicians, with their depth of training and experience, remain indispensable in interpreting LLM-generated content, molding it to the unique contours of each patient's clinical situation. In weaving LLMs into the fabric of healthcare, the aim should be to augment the patient-physician relationship, fortifying the principle of informed decision-making and ensuring that patients continue to be at the center of their care trajectory.

Table 2 presents both the potential benefits and crucial considerations when incorporating LLMs into SDM. It emphasizes the need to weigh advantages against ethical and practical concerns.

**Table 2:** Overview of implementing LLMs in SDM for healthcare

Category	Sub-category	Details	Implications/strategies
Benefits of LLMs	Accessible information	Provides tailored information, addressing patient concerns and queries, enhancing understanding and engagement	Ensures information is aligned with patient needs and preferences, complementing clinical expertise
	Personalized care	LLMs can analyze vast datasets to offer personalized insights and recommendations for patient care	Tailor healthcare strategies to individual patient profiles, improving outcomes and patient satisfaction
	Support for clinical decision-making	Augments clinician knowledge with evidence-based suggestions, reducing diagnostic and therapeutic errors	Enhance the accuracy of clinical decisions, support complex case analysis, and complement clinician expertise
	Patient education and empowerment	Facilitates deeper patient understanding of their health conditions and treatment options	Empower patients to actively participate in their care, leading to better health outcomes and satisfaction
	Clinical training and education	Offers a dynamic, interactive platform for medical education and professional development	Improve the training of healthcare professionals with up-to-date, scenario-based learning tools
	Research and development	Accelerates medical research by analyzing patterns and generating hypotheses from large datasets	Drive innovation in treatment methods, diagnostic tools, and healthcare delivery models
	Efficiency in healthcare operations	Streamlines administrative tasks, patient triage, and resource allocation	Optimize healthcare delivery, reduce wait times, and improve overall operational efficiency
Considerations for LLM use	Ethical and privacy concerns	Ethical considerations for patient data privacy and consent	Integration with healthcare workflows, responsible AI use, ongoing evaluation, and literacy level adjustments in responses
	Integration and workflow alignment	Integration with existing healthcare workflows and practices	Align tools with patient and clinical needs, facilitating seamless engagement and complementing expertise
Challenges in SDM	Accuracy and reliability	AI-generated information may not always be accurate or up-to-date	Decision-making may rely on outdated information, necessitating continuous updates and accuracy checks
	Interpretability	Complexity in AI outputs can hinder patient and clinician understanding	Develop intuitive interfaces and present information in lay terms for effective communication
	Bias and equity	Perpetuation of biases affects fairness in recommendations	Implement diverse datasets and continuous auditing to mitigate biases and ensure equitable care
	Data privacy	Concerns over data security and patient privacy	Enhance data protection measures and communicate practices transparently to build trust
Technical requirements	Computational resources	Necessity for high-performance computing for LLM operations	Ensure availability of resources to support large-scale data processing and AI computations
	Data infrastructure	Secure, compliant infrastructure for patient data management	Robust data protection and privacy measures are crucial for secure and efficient data handling
	Integration capabilities	Compatibility with EHRs and clinical decision support systems	Facilitate seamless integration to leverage existing digital health infrastructures
	User interface and training	Development of user-friendly interfaces and ongoing professional education	Enhance interaction with LLM outputs and ensure effective use and interpretation in clinical settings
Strategies for overcoming limitations	Algorithmic bias	Bias leading to unequal care outcomes	Diverse training datasets and continuous auditing to identify and address biases
	Data privacy concerns	Impacts patient trust and data sharing willingness	Strengthen data protection and communicate handling practices clearly
	Real-time updates	AI recommendations may not reflect the latest research	Incorporate mechanisms for continuous learning and updates based on new data and studies
	Interdisciplinary collaboration	Essential but often lacking for effective implementation	Foster teams of clinicians, AI experts, ethicists to guide development and deployment
	Regulatory and ethical considerations	Navigating the complex regulatory landscape and ethical dilemmas	Develop guidelines and ethical frameworks, engaging with regulatory bodies and ethicists early in the process

This table delineates the multifaceted considerations associated with the implementation and utilization of LLMs within the context of SDM in healthcare settings. It systematically categorizes critical aspects into five main areas: the benefits of employing LLMs for enhancing patient engagement and understanding; the considerations necessary for ethical deployment and integration into existing clinical workflows; the challenges faced in ensuring the accuracy, interpretability, and equity of AI-generated information; the technical and infrastructural requirements for effective LLM implementation; and strategic approaches for overcoming potential limitations such as algorithmic bias, privacy concerns, and the need for real-time updates. Each category and sub-category is elaborated with details on the specific issue or benefit, alongside implications or strategies aimed at addressing these aspects to optimize patient care outcomes and support healthcare professionals

*AI* artificial intelligence, *LLM* large language model, *SDM* shared decision-making, *EHR* electronic health record

## Future directions

The field of neuro-oncology, characterized by its significant emotional and cognitive demands on patients, presents a distinctive context for decision-making processes (Pertz et al. 2022). This context positions neuro-oncology at the intersection of clinical challenges and technological opportunities, particularly regarding the implementation of LLMs within SDM frameworks. The future trajectory of LLMs in SDM is both promising and complex, necessitating strategic planning for their integration.

Central to this integration is the imperative for ethical considerations. Developing clear ethical guidelines and regulatory frameworks for LLM technologies in healthcare is critical. Such frameworks must address a range of concerns, including patient data privacy, transparency, and informed consent, alongside efforts to mitigate biases and promote responsible use (Meskó and Topol 2023). These elements are essential for establishing a strong ethical foundation for the application of LLMs in neuro-oncology.

The utility of ethically designed LLM tools is contingent upon their compatibility with clinical workflows. Future developments should focus on integrating LLM tools seamlessly into clinical practices, which can be facilitated by designing intuitive user interfaces, ensuring compatibility with existing electronic health records, and creating synergies with current decision-support systems (Sallam 2023a). Effective integration is key to optimizing the utility of LLMs and securing their acceptance within the medical community (Sallam 2023b). Moreover, the focus on patient-centric outcomes is vital. Investigating the effects of LLMs on patient satisfaction, decision quality, treatment adherence, and long-term health outcomes is crucial for assessing the real-world impact of LLMs in SDM.

Furthermore, the education and training of healthcare professionals regarding LLM technologies are of paramount importance. Ensuring that clinicians are knowledgeable about the capabilities, limitations, and potential biases of LLMs is necessary for informed clinical decision-making and maintaining trust between healthcare providers and patients.

Finally, equipping the current and next generation of healthcare professionals is paramount (Abd-Alrazaq et al. 2023). The integration of LLMs in neuro-oncology necessitates specialized education and training endeavors. By ensuring clinicians are well-versed in the strengths, pitfalls, and nuanced biases of LLM technologies, we can pave the way for more informed clinical decisions and reinforce the mutual trust between healthcare providers and their patients (Cascella et al. 2023).

## Conclusion

SDM appears to offer significant potential in neuro-oncology care. As efforts are made to foster a more collaborative environment between patients and healthcare professionals, LLMs might be considered as tools to support patient education, counseling, and initial dialogues. They might aid in delivering information, addressing patient queries, and enhancing clarity regarding treatment choices, which could, in turn, help patients feel more involved, possibly improve patient-doctor interactions, and alleviate some disease and treatment-related anxiety and apprehensions.

Integrating LLMs with SDM practices offers the opportunity to increase patient engagement, potentially improve treatment outcomes, and foster a more supportive relationship between patients and doctors in neuro-oncology care. However, it is essential to recognize that LLMs are supplements, not substitutes, for clinical expertise. Their potential to enhance the decision-making process with accessible and personalized information must therefore be approached with careful consideration of ethical standards, data protection, and continuous evaluation.

## Data Availability

All datasets generated and analyzed during the current study are included in this published article. No additional data are available.

## References

[CR1] Abd-Alrazaq A, Alsaad R, Alhuwail D (2023). Large language models in medical education: opportunities, challenges, and future directions. JMIR Med Educ.

[CR2] Baumgartner C, Baumgartner D (2023). A regulatory challenge for natural language processing (NLP)-based tools such as ChatGPT to be legally used for healthcare decisions. where are we now?. Clin Transl Med.

[CR3] Benjamens S, Dhunnoo P, Meskó B (2020). The state of artificial intelligence-based FDA-approved medical devices and algorithms: an online database. Npj Digit Med.

[CR4] Bommasani R, Liang P, Lee T (2023). Holistic evaluation of language models. Ann N Y Acad Sci.

[CR5] Borger JG, Ng AP, Anderton H (2023). Artificial intelligence takes center stage: exploring the capabilities and implications of ChatGPT and other AI-assisted technologies in scientific research and education. Immunol Cell Biol.

[CR6] Jesserun CAC, Broekman MLD (2022). True shared decision-making in neurosurgical oncology: does it really exist?. Acta Neurochir.

[CR7] Cascella M, Montomoli J, Bellini V (2023). Evaluating the feasibility of ChatGPT in healthcare: an analysis of multiple clinical and research scenarios. J Med Syst.

[CR8] Chieffo DPR, Lino F, Ferrarese D (2023). Brain tumor at diagnosis: from cognition and behavior to quality of life. Diagnostics.

[CR9] Coomans MB, Van Der Linden SD, Gehring K (2019). Treatment of cognitive deficits in brain tumour patients: current status and future directions. Curr Opin Oncol.

[CR10] Corell A, Guo A, Vecchio TG (2021). Shared decision-making in neurosurgery: a scoping review. Acta Neurochir.

[CR11] Dash D, Thapa R, Banda JM et al. (2023) Evaluation of GPT-3.5 and GPT-4 for supporting real-world information needs in healthcare delivery. In, p arXiv:2304.13714

[CR12] Díaz JL, Barreto P, Gallego JM (2009). Proper information during the surgical decision-making process lowers the anxiety of patients with high-grade gliomas. Acta Neurochir.

[CR13] Dinan E, Abercrombie G, Stevie Bergman A et al. (2021) Anticipating safety issues in E2E conversational AI: framework and tooling. In, p arXiv:2107.03451

[CR14] Downing NL, Bates DW, Longhurst CA (2018). Physician burnout in the electronic health record era: are we ignoring the real cause?. Ann Int Med.

[CR15] Dunbar EM, Kumthekar PU (2020). In pursuit of a perpetually burning flame: preventing burnout in neuro-oncology. Neuro Oncol.

[CR16] Edwards M, Holland-Hart D, Mann M (2023). Understanding how shared decision-making approaches and patient aids influence patients with advanced cancer when deciding on palliative treatments and care: a realist review. Health Expect.

[CR17] Elwyn G, Frosch DL, Kobrin S (2016). Implementing shared decision-making: consider all the consequences. Implement Sci.

[CR18] Gao A (2023). Prompt engineering for large language models. SSRN Electr J.

[CR19] Gerstenecker A, Nabors LB, Meneses K (2014). Cognition in patients with newly diagnosed brain metastasis: profiles and implications. J Neurooncol.

[CR20] Gosselt IK, Scheepers VPM, Spreij LA (2021). Cognitive complaints in brain tumor patients and their relatives’ perspectives. Neuro-Oncol Pract.

[CR21] Harrer S (2023). Attention is not all you need: the complicated case of ethically using large language models in healthcare and medicine. EBioMedicine.

[CR22] Haver HL, Gupta AK, Ambinder EB (2024). Evaluating the use of ChatGPT to accurately simplify patient-centered information about breast cancer prevention and screening. Radiol Imag Cancer.

[CR23] Hermann H, Trachsel M, Elger BS (2016). Emotion and value in the evaluation of medical decision-making capacity: a narrative review of arguments. Front Psychol.

[CR24] Hewins W, Zienius K, Rogers JL (2019). The effects of brain tumours upon medical decision-making capacity. Curr Oncol Rep.

[CR25] Heyhoe J, Birks Y, Harrison R (2015). The role of emotion in patient safety: are we brave enough to scratch beneath the surface?. J R Soc Med.

[CR27] Hong J, Wang JT, Zhang C et al. (2023) DP-OPT: make large language model your privacy-preserving prompt engineer. in, p arXiv:2312.03724

[CR28] Huang L, Yu W, Ma W et al. (2023) A survey on hallucination in large language models: principles, taxonomy, challenges, and open questions. in, p arXiv:2311.05232

[CR29] Hussien A-E-EA, Hamza N, Hefny HA (2013). Attacks on anonymization-based privacy-preserving: a survey for data mining and data publishing. J Inf Secur.

[CR30] Jin D, Pan E, Oufattole N (2021). What disease does this patient have? a large-scale open domain question answering dataset from medical exams. Appl Sci.

[CR31] Kaba R, Sooriakumaran P (2007). The evolution of the doctor-patient relationship. Int J Surg.

[CR32] Koch-Weser S, Dejong W, Rudd RE (2009). Medical word use in clinical encounters. Health Expect.

[CR33] Kroth PJ, Morioka-Douglas N, Veres S (2019). Association of electronic health record design and use factors with clinician stress and burnout. JAMA Netw Open.

[CR34] Lee P, Bubeck S, Petro J (2023). Benefits, limits, and risks of GPT-4 as an AI chatbot for medicine. N Engl J Med.

[CR35] Légaré F, Adekpedjou R, Stacey D (2018). Interventions for increasing the use of shared decision making by healthcare professionals 2018. Cochrane Datab Syst Rev.

[CR36] Leu S, Cahill J, Grundy PL (2023). A prospective study of shared decision-making in brain tumor surgery. Acta Neurochir.

[CR37] Li J, Dada A, Puladi B, Kleesiek J, Egger J (2024). ChatGPT in healthcare: a taxonomy and systematic review. Comput Methods Programs Biomed.

[CR38] Liao QV, Wortman Vaughan J (2023) AI Transparency in the Age of LLMs: A Human-Centered Research Roadmap. In, p arXiv:2306.01941

[CR39] Ling C, Zhao X, Lu J et al. (2023) Domain specialization as the key to make large language models disruptive: a comprehensive survey. In, p arXiv:2305.18703

[CR40] Lukas RV, Wu J, Dey M (2018). A survey of the neuro-oncology landscape. J Clin Neurol.

[CR41] Makoul G, Clayman ML (2006). An integrative model of shared decision making in medical encounters. Patient Educ Couns.

[CR42] Meskó B, Topol EJ (2023). The imperative for regulatory oversight of large language models (or generative AI) in healthcare. Npj Digit Med.

[CR43] De Mik SML, Stubenrouch FE, Balm R (2018). Systematic review of shared decision-making in surgery. Br J Surg.

[CR44] Mittelstadt B, Wachter S, Russell C (2023). To protect science, we must use LLMs as zero-shot translators. Nat Hum Behav.

[CR45] Mökander J, Schuett J, Kirk HR (2023). Auditing large language models: a three-layered approach. AI Eth.

[CR46] Musella A, Devitto R, Anthony M (2021). The Importance of shared decision-making for patients with glioblastoma. Patient Prefer Adher.

[CR47] Nasr M, Carlini N, Hayase J et al. (2023) Scalable extraction of training data from (production) language Models. In, p arXiv:2311.17035

[CR48] Onder CE, Koc G, Gokbulut P (2024). Evaluation of the reliability and readability of ChatGPT-4 responses regarding hypothyroidism during pregnancy. Sci Rep.

[CR49] Pace A, JaF K, Van Den Bent MJ (2020). Determining medical decision-making capacity in brain tumor patients: why and how?. Neuro-Oncol Pract.

[CR50] Pertz M, Schlegel U, Thoma P (2022). Sociocognitive functioning and psychosocial burden in patients with brain tumors. Cancers.

[CR51] Porter AB, Chukwueke UN, Mammoser AG (2021). Delivering equitable care to underserved neuro-oncology populations. Am Soc Clin Oncol Educ B.

[CR52] Ray PP (2023). ChatGPT: a comprehensive review on background, applications, key challenges, bias, ethics, limitations and future scope. Intern Th Cyber-Phys Syst.

[CR53] Reinert C, Rathberger K, Klinkhammer-Schalke M (2018). Information needs and requirements in patients with brain tumours and their relatives. J Neurooncol.

[CR54] Roberts K, Dowell A, Nie JB (2019). Attempting rigour and replicability in thematic analysis of qualitative research data; a case study of codebook development. BMC Med Res Methodol.

[CR55] Sallam M (2023). ChatGPT utility in healthcare education, research, and practice: systematic review on the promising perspectives and valid concerns. Healthcare.

[CR56] Shepherd SC, Hacking B, Wallace LM (2023). Feeling known and informed: Serial qualitative interviews evaluating a consultation support intervention for patients with high-grade glioma. Cancer Med.

[CR57] Singhal K, Azizi S, Tu T (2023). Large language models encode clinical knowledge. Nature.

[CR58] Sorensen Von Essen H, Poulsen FR, Dahlrot RH (2022). Development of a patient decision aid to support shared decision making for patients with recurrent high-grade glioma. Int J Environ Res Pub Health.

[CR59] Sorensen Von Essen H, Stacey D, Dahl Steffensen K (2022). Decisional needs of patients with recurrent high-grade glioma and their families. Neurooncol Pract.

[CR60] Temel MH, Erden Y, Bağcıer F (2024). Information quality and readability: ChatGPT’s responses to the most common questions about spinal cord injury. W Neurosurg.

[CR61] Topol EJ (2019). High-performance medicine: the convergence of human and artificial intelligence. Nat Med.

[CR62] Vermeir P, Vandijck D, Degroote S (2015). Communication in healthcare: a narrative review of the literature and practical recommendations. Int J Clin Pract.

[CR63] Waddell A, Lennox A, Spassova G (2021). Barriers and facilitators to shared decision-making in hospitals from policy to practice: a systematic review. Implement Sci.

[CR64] Wang C, Ong J, Wang C (2023). Potential for GPT technology to optimize future clinical decision-making using retrieval-augmented generation. Ann Biom Eng.

[CR65] West CP, Dyrbye LN, Shanafelt TD (2018). Physician burnout: contributors, consequences and solutions. J Intern Med.

[CR66] Yang R, Tan TF, Lu W (2023). Large language models in health care: development, applications, and challenges. Health Care Sci.

[CR67] Yust-Katz S, O’brienVera BE (2020). Burnout and career satisfaction in neuro-oncology: a survey of the society for neuro-oncology and the european association of neuro-oncology memberships. Neuro Oncol.

[CR26] Zakka C, Shad R, Chaurasia A, Dalal AR, Kim JL, Moor M, Fong R, Phillips C, Alexander K, Ashley E, Boyd J, Boyd K, Hirsch K, Langlotz C, Lee R, Melia J, Nelson J, Sallam K, Tullis S, Vogelsong MA, Cunningham JP, Hiesinger W (2024) Almanac - retrieval-augmented language models for clinical medicine. NEJM AI 1(2). 10.1056/aioa2300068 10.1056/aioa2300068 PMC10857783 38343631

[CR68] Zhang S, Ray S, Lu R (2022). Toward privacy-preserving aggregate reverse skyline query with strong security. IEEE Trans Inf Forens Secur.

